# Advances and Challenges in Vaccination and Therapeutic Strategies Against Japanese Encephalitis Virus

**DOI:** 10.3390/pathogens14121204

**Published:** 2025-11-26

**Authors:** Jae-Yeon Park, Hye-Mi Lee

**Affiliations:** College of Veterinary Medicine, Chungnam National University, Daejeon 34134, Republic of Korea

**Keywords:** Japanese encephalitis virus, vaccination, therapeutic strategies, genotype diversity, public health, One Health, cross-genotype neutralization

## Abstract

The Japanese encephalitis virus (JEV) remains a major cause of viral encephalitis in Asia, and recent epidemiological shifts driven by the predominance of genotype I and the re-emergence of genotype V have renewed concerns regarding control efforts. Licensed vaccines have a reduced incidence of more than 90% in several endemic regions; however, evidence of reduced cross-neutralization against heterologous genotypes indicates that vaccines derived from genotype III strains may not fully match the evolving antigenic landscape. This review synthesizes current knowledge on vaccine performance, genotype-driven antigenic variation, and implications for future strain alignment. Emerging platforms, including mRNA, DNA, virus-like particles, and structure-guided recombinant antigens, have been evaluated for their potential to enhance cross-genotype breadth, scalability, and thermostability. We also summarize the progress in antiviral discovery targeting viral nonstructural proteins, host pathways, and monoclonal antibody development, along with immunomodulatory and neuroprotective strategies. Translational challenges, such as blood–brain barrier penetration, therapeutic timing, and durability of immunity, have been highlighted as key barriers to clinical application. By integrating molecular, immunological, and epidemiological evidence, this review outlines strategic directions for developing broad-spectrum vaccines and therapeutics capable of addressing the evolving genetic and ecological landscape of JEV.

## 1. Introduction

Japanese encephalitis virus (JEV) is a mosquito-borne flavivirus that is the leading cause of viral encephalitis in Asia, with an estimated 68,000 cases and 10,000–20,000 deaths annually [1,2]. More than three billion people live in endemic or at-risk regions in Southeast Asia, the Western Pacific, and Northern Australia [2,3]. The disease predominantly affects children and immunologically naïve adults, with clinical outcomes ranging from mild febrile illness to severe encephalitis, high fatality rates, and long-term neurological sequelae in survivors [4,5].

Over the past five decades, large-scale vaccination programs have markedly reduced the incidence of JEV in several endemic countries [6]. Inactivated vaccines derived from genotype III strains, including Nakayama and Beijing-1, form the basis of the early national immunization programs [7]. The live-attenuated SA14-14-2 vaccine has demonstrated excellent protective efficacy and has been widely adopted across Asia [8,9]. Recently, Vero cell-derived inactivated and chimeric vaccines have expanded the global immunization options [10,11]. Despite these advances, gaps remain in cross-genotype protection, long-term immunity in specific age groups, and accessibility in resource-limited settings [12,13].

Molecular surveillance has revealed substantial genotype shifts with direct implications for vaccine performance. Genotype I (GI) has progressively displaced genotype III (GIII) across East and Southeast Asia [14,15], and genotype V (GV) has re-emerged in China and the Republic of Korea after decades of apparent absence [16,17]. Multiple studies have reported reduced neutralizing antibody titers against GI and especially GV when sera from recipients of GIII-based vaccines were tested in plaque-reduction neutralization assays [13,18]. These genotype-associated antigenic differences raise concerns that continued reliance on vaccines developed against GIII strains may be insufficient to address the evolving viral landscape of GIII rotaviruses in humans.

Currently, there is no licensed antiviral therapy for acute Japanese encephalitis (JE). Although numerous small-molecule inhibitors, repurposed antivirals, and monoclonal antibodies have demonstrated antiviral activity in vitro and in animal models [19,20], most remain at the preclinical or early translational stages, and their ability to achieve therapeutic concentrations in the central nervous system remains uncertain [20,21]. This translational gap highlights the need for integrated antiviral, immunomodulatory, and neuroprotective strategies to combat this disease.

To address these challenges, this study focused on three central themes. First, we summarize how recent genotype shifts challenge existing GIII-derived vaccines and evaluate evidence for reduced cross-genotype neutralization. Second, we outline the development of next-generation vaccine platforms, including virus-like particles (VLPs), recombinant subunit vaccines, nucleic acid vaccines, genotype-matched constructs, and approaches designed to enable broad cross-genotype protection [10,22,23]. Third, we review the progress in therapeutic strategies and discuss the key factors that have slowed their translation into clinical applications. By integrating epidemiological, molecular, immunological, and therapeutic data, this review provides an updated framework for understanding the strengths and limitations of current interventions and the priorities required to achieve broad and durable control of Japanese encephalitis in the context of emerging genotypes [15,24,25]. This review focuses on how the recent dominance of GI, GIV, and GV challenges the antigenic match of vaccines derived from GIII. It also compares the strategies proposed by next-generation platforms that aim to overcome these genotype-related limitations. Finally, it summarizes the factors that have slowed the translational progress of antiviral therapeutics and outlines the directions needed to advance these candidates for clinical application.

## 2. JEV Biology and Immune Correlates

### 2.1. Virus Structure and Genome Organization

JEV is an enveloped, positive-sense, single-stranded RNA virus of the genus *Flavivirus*, measure approximately 50 nm in diameter [26]. The genomic RNA (~11 kb) encodes a single open reading frame flanked by untranslated regions (UTRs) containing conserved RNA structures essential for replication [27,28]. Structural proteins (capsid [C], precursor membrane [prM/M], and envelope [E]) and seven nonstructural proteins (NS1, NS2A, NS2B, NS3, NS4A, NS4B, and NS5) were generated through co- and post-translational processing [28,29]. The E protein mediates receptor binding and membrane fusion and is the principal target of neutralizing antibodies, whereas NS1 contributes to immune evasion and endothelial permeability [30,31]. Recent structural studies using cryo-electron microscopy have refined our understanding of virion organization and highlighted the conformational epitopes relevant for neutralizing antibody responses and vaccine design [32].

### 2.2. Replication and Pathogenesis

Following receptor-mediated endocytosis, acid-dependent fusion releases viral RNA into the cytoplasm, where translation of the polyprotein and replication occur on endoplasmic reticulum-derived membranes [31,33,34]. JEV disseminates to peripheral tissues before entering the central nervous system (CNS), facilitated in part by cytokine-driven disruption of blood–brain-barrier (BBB) integrity [35,36,37]. Infection results in neuronal apoptosis, microglial activation, and cytokine release, which contribute to the pathology [35,38,39]. These mechanisms form the foundation for the subsequent discussion of neuroinflammation-targeted therapeutic approaches.

### 2.3. Host Immune Responses

Innate immune sensing through receptors, including Toll-like receptor 3, retinoic acid-inducible gene I (RIG-I), and MDA5, induces interferon-mediated antiviral defenses [40,41,42]. JEV limits these defenses by promoting signal transducer and activator of transcription 2 (STAT2) degradation and disrupting RIG-I–MAVS signaling through several nonstructural proteins [41,43,44]. These early pathways also influence inflammatory responses that contribute to neurological injury, and this link is examined further in Section 7.

Adaptive immunity supports viral clearance and provides long-term protection. Neutralizing antibodies targeting the E protein represent an established correlation of protection and form the basis of vaccine-mediated immunity [40,45,46]. CD4^+^ T cells assist in humoral responses, and CD8^+^ cytotoxic T cells eliminate infected cells [21,47].

### 2.4. Correlates of Protection

Neutralizing antibodies measured by plaque-reduction neutralization tests (PRNTs) remain the primary correlate of protection, with a PRNT_50_ ≥ 1:10 widely accepted as protective [48,49]. Additional immune components, including memory B cells, long-lived plasma cells, and T cell responses, contribute to durable immunity [47,50]. Antibody titers may decline over time, especially in children and older adults, which has implications for booster scheduling [49,51]. Standardized PRNT protocols recommended by the World Health Organization help mitigate interlaboratory variability in serological evaluations [52].

### 2.5. Cross-Reactivity and ADE (Antibody-Dependent Enhancement)

Flaviviruses share substantial antigenic similarities, resulting in cross-reactive antibody responses among JEV, DENV, and ZIKV [12,53]. Depending on their affinity and concentration, cross-reactive antibodies can mediate either neutralization or enhancement via Fc receptor-dependent uptake [54,55]. Although ADE have been demonstrated in vitro and in several animal models, evidence for clinically relevant ADE in human JEV infection or vaccination remains limited. Epitope specificity and cross-reactive antibody responses are crucial for shaping the breadth of vaccine-induced immunity. These factors are particularly relevant for evaluating next-generation vaccine platforms designed to improve cross-genotype protection. The concern regarding antibody-dependent enhancement in the context of JEV vaccination is largely theoretical and is based mainly on in vitro findings and experience from dengue virus. Consistent with previous studies, clinically confirmed cases of vaccine-associated enhancement are extremely rare, and large-scale field studies have not shown increased disease severity among vaccinated individuals. ADE remains a consideration in flavivirus-endemic settings, but the current evidence indicates that the actual risk for JEV vaccines is very low and should not be overstated.

## 3. Epidemiology, Transmission Dynamics, and Genotype Distribution

### 3.1. Geographic Distribution and Changing Epidemiological Patterns

Recent genotype displacement has increased concern that the growing dominance of GI and the reappearance of genotype V may weaken the alignment between circulating viruses and the GIII strains that form the basis of all licensed vaccines. This genotype displacement pressure is the central theme of this review and guides the subsequent discussions on cross-genotype neutralization and the need for updated vaccine platforms. JEV remains endemic to South Asia, East Asia, and parts of Oceania [2]. Historically, GIII has been predominant in most endemic regions for several decades. However, recent surveillance has revealed geographic expansion into regions previously considered non-endemic, including northern Australia and parts of the Western Pacific [56].

Environmental and ecological changes have contributed to the shifting transmission patterns. Climate-driven changes in temperature, rainfall, and vector distribution, along with intensified pig farming and rice irrigation, have altered mosquito breeding habitats, and may facilitate the spread of viruses to new areas [52,57]. Despite progress in vaccination, significant heterogeneity persists across countries, with rural agricultural communities being the most affected.

### 3.2. Genotype Shifts and Emerging Strains

JEV is classified into five major genotypes based on E gene phylogeny. Over the past two decades, GI has progressively replaced GIII across East and Southeast Asia [58,59]. This displacement has been documented in China, Japan, Korea, India, and Vietnam, suggesting that GI strains possess ecological or vector-related advantages [14,59].

GV, which has rarely been detected, has re-emerged in China and the Republic of Korea [60,61], raising concerns because of its greater antigenic divergence relative to GIII vaccine strains. Although the biological basis of genotype displacement is not fully understood, hypotheses include differences in viral replication efficiency, vector competence, and climatic suitability for GI-associated mosquito populations [62,63].

### 3.3. Implications of Genotype Shifts for Vaccine Effectiveness

Multiple serological studies have shown that sera from recipients of GIII-based vaccines exhibit a two-fold reduction in neutralizing antibody titers against genotype I and, more markedly, against GV strains [64,65]. Despite this reduction in in vitro neutralization, field data still support strong protection against severe disease, particularly GI infections [66,67]. However, the antigenic distance between GIII vaccine strains and emerging genotypes has increased, and breakthrough infections may become more likely if genotype V continues to expand geographically.

Genotype-associated variations in epitopes that form quaternary neutralizing determinants of E protein may contribute to reduced cross-neutralization. Differences in domain III residues and structural flexibility of the E dimer interface have been proposed as the mechanisms underlying these patterns [65,68]. These findings underscore the need to evaluate whether existing vaccines provide adequate protection in regions experiencing rapid genotype turnover. Continued molecular surveillance is essential for tracking genotype movement and antigenic drift, and these data will help guide decisions regarding vaccine strain selection and cross-genotype neutralization assessment.

## 4. Licensed Vaccines

### 4.1. Historical Development of Licensed Vaccines

The development of JE vaccines has been a major success in the field of arboviral diseases. First-generation vaccines, produced in the 1940s and the 1950s, consist of formalin-inactivated mouse-brain-derived preparations generated from GIII strains, such as Nakayama and Beijing-1 [69,70]. These vaccines achieved high protective efficacy but exhibited batch variability and rare hypersensitivity reactions related to residual neural tissue components [71]. Despite these limitations, they have been deployed in Japan, Taiwan, and the Republic of Korea for more than five decades and have contributed to a >99% reduction in JE incidence [72,73].

To overcome safety and production limitations, second-generation inactivated vaccines have been developed using the Vero cell culture technology. The GIII-based inactivated formulations derived from SA14-14-2, including IXIARO^®^ and JESPECT^®^, offer improved manufacturing consistency, enhanced safety, and international licensure [74,75]. These non-mouse-brain-derived vaccines were prequalified by the WHO in 2013 and are globally recommended for both travelers and endemic populations [76,77].

The live-attenuated SA14-14-2 vaccine, developed in China in 1988, also originated from a GIII strain and remains the cornerstone of JE vaccination programs in Asia. It has been administered to hundreds of millions of children with excellent safety and greater than 95% protective efficacy [7]. This vaccine elicits durable neutralizing antibodies and memory T cell responses and has been integrated into the national immunization schedules in China, India, Nepal, Sri Lanka, and Vietnam [8].

Chimeric live vaccines incorporating GIII-derived SA14-14-2 prM/E genes into a YF-17D or attenuated dengue backbone provide additional options. The ChimeriVax-JE (IMOJEV^®^) vaccine is licensed in Australia, Thailand, and the Philippines and induces strong immunogenicity after a single dose [78,79]. The live-attenuated JE-CV formulation demonstrated non-inferior immunogenicity compared to traditional SA14-14-2 [49]. Across these licensed platforms, a common feature is their origin from GIII strains, which have historically matched globally circulating viruses. However, recent genotype shifts have raised the question of whether these GIII-based vaccines will retain optimal coverage in the future.

### 4.2. Comparative Immunogenicity and Efficacy of Licensed Vaccines

All licensed JE vaccines generate robust neutralizing antibody responses, surpassing the accepted protective PRNT_50_ threshold of 1:10 in >95% of recipients following primary immunization [75]. Inactivated Vero cell-derived vaccines, such as IXIARO^®^ and JESPECT^®^, achieve seroconversion rates of 96–100% after two doses (days 0 and 28) in both adults and children [75,80]. Neutralizing antibodies generally persist for 12–24 months, and booster doses effectively restore waning titers [51,81]. Adverse events are usually mild and include brief injection-site discomfort and transient fatigue [82].

The live-attenuated SA14-14-2 vaccine induces ≥ 95% seroconversion within four weeks of a single dose, with a vaccine effectiveness of 96–99% reported in China, Nepal, and India [8,83]. Live vaccines typically elicit stronger neutralizing and cellular immunities than inactivated formulations [84,85].

Chimeric vaccines, such as IMOJEV^®^ and JE-CV, demonstrate non-inferior immunogenicity and safety, comparable to that of SA14-14-2. A randomized trial reported 99% seroconversion within 28 days, with titers persisting above the protective thresholds for at least five years [86,87]. Although platform differences influence immunogenicity, all licensed vaccines share a GIII origin and maintain broad protection against GI and GIV in field evaluations [87,88]. Given the ongoing shift toward GI dominance and the re-emergence of GV, sustained post-marketing surveillance is increasingly important to ensure that licensed GIII-derived vaccines continue to provide adequate cross-genotype protection. To further clarify the genotype-specific performance of licensed GIII-derived vaccines, the reported reductions in neutralization titers and available field or experimental evidence are summarized in Table 1. A four-fold or greater reduction in PRNT titers is generally considered meaningful when evaluating antigenic differences among JEV genotypes.

### 4.3. Safety Profiles and Adverse Events

The safety profiles of licensed JE vaccines are well established and supported by decades of clinical and post-marketing evidence [69,88] (Table 2). Although effective, first-generation mouse-brain-derived vaccines are associated with higher rates of systemic reactions, such as fever, headache, and rare neurological complications, including acute disseminated encephalomyelitis and Guillain–Barré syndrome (approximately 1–2 per million doses) [89]. These concerns have contributed to their gradual replacement with cell culture-based vaccines.

Vero cell-derived inactivated vaccines (IXIARO^®^ and JESPECT^®^) have shown markedly improved safety. Large trials have reported mild injection-site pain (20–30%), transient fatigue (10–15%), and low-grade fever (≤5%) to be the most frequent effects [69]. Importantly, no vaccine-related serious adverse events or neurological complications have been causally linked to these modern inactivated formulations [90,91].

The live-attenuated SA14-14-2 vaccine has been administered to >400 million individuals and has exhibited exceptional tolerability. Post-marketing reviews from Asia reported only transient fever (5–10%) and mild rash (<1%), with no evidence of neurotropism or viscerotropism [92]. WHO-sponsored reviews indicate that SA14-14-2 is among the safest, live viral vaccines [69].

Chimeric vaccines (IMOJEV^®^ and JE-CV) have similar favorable safety profiles. Across studies involving more than 3000 participants, no vaccine-related serious adverse events were documented, and the symptoms were limited to mild local reactions or transient myalgia [93]. Despite their YF vector backbone, no cases of vaccine-associated viscerotropic or neurotropic diseases have been reported [94]. Overall, licensed JE vaccines exhibit excellent safety-to-benefit ratios. Continued pharmacovigilance remains important, particularly in regions undergoing genotype shifts where cross-reactive immunity or age-specific responses may influence vaccine performance.

**Table 2:** Licensed JEV vaccines and their genotypes.

Vaccine Type	Representative Product (Strain)	Platform/Production	Dosing Schedule	Immunogenicity/Duration	WHO Status/Region	Genotype Origin	Ref.
Inactivated mouse-brain	Nakayama, Beijing-1	Formalin-inactivated, mouse-brain derived	3-dose (0, 7, 28 days)	Seroconversion 95–100%; protection ≈ 2–3 years	Historical use (Japan, Korea, Taiwan)	GIII	[95,96]
Inactivated Vero-cell	IXIARO^®^, JESPECT^®^ (Beijing-1/SA14-14-2)	Vero-cell culture	2-dose (0, 28 days) + booster (12 months)	96–100% seroconversion; GMTs sustained 12–24 months	WHO-prequalified; global use	GIII	[81,97,98]
Live-attenuated	SA14-14-2	Primary hamster kidney cell culture	Single dose	≥95% seroconversion; ≥5 years protection	China, India, Nepal, Vietnam, SE Asia programs	GIII	[7,88,99]
Chimeric (YF 17D backbone)	IMOJEV^®^, JE-CV	Recombinant YF-17D vector expressing SA14-14-2 prM–E	Single dose	≈99% seroconversion; ≥5 years immunity	Australia, Thailand, Philippines	GIII	[100,101,102]

GMT, geometric mean titer; prM–E, precursor membrane and envelope proteins; YF-17D, yellow fever 17D vaccine strain.

## 5. Emerging Vaccine Strategies

### 5.1. Novel Platforms and Preclinical Candidates

The reduced neutralization against GI and GV observed in several studies explains why new platforms are being evaluated to maintain broad protection as genotype patterns continue to change. Although licensed JE vaccines remain highly effective against severe diseases, recent genotype shifts, including widespread GI dominance and the re-emergence of GV, highlight the limitations of vaccines derived exclusively from the GIII strains [88]. Therefore, emerging platforms aim to improve cross-genotype breadth, reduce dependence on complex manufacturing systems, and enable rapid antigen updates in response to viral evolution [103,104].

mRNA vaccines, which have been validated during the COVID-19 pandemic, are being actively explored for JEV immunization [105]. Prototype constructs encoding JEV E or EDIII encapsulated in lipid nanoparticles elicited strong neutralizing antibodies and balanced Th1 and Th2 responses in mice and swine [106]. Notably, an mRNA vaccine expressing a codon-optimized GI E protein achieved complete protection in mice [107], illustrating the platform’s suitability for rapid adaptation to genotype-replacement events.

DNA vaccines encoding the prM and E antigens under the cytomegalovirus promoter provide protective immunity in animal models. The incorporation of molecular adjuvants, such as IL-2 or granulocyte–macrophage colony-stimulating factor, enhances T-cell responses [108]. However, neutralizing antibody titers remain lower than those elicited by live-attenuated vaccines, and delivery improvements, including electroporation or nanoparticle carriers, may be required to achieve broader cross-genotype coverage [109].

Virus-like particle vaccines generated using baculovirus or mammalian expression systems contain native quaternary E protein epitopes and induce complete protection in mouse challenge models [110]. Because VLPs preserve conformational epitopes that differ among genotypes, this platform may facilitate improved cross-genotype neutralization, including that against more antigenically divergent strains, such as GV [111].

Recombinant viral vector vaccines, including adenovirus-, vaccinia-, and measles-based constructs, generate robust humoral and cellular responses in preclinical studies [112]. Such vectors may also serve as heterologous boosters to broaden immunity in populations primed with inactivated or live-attenuated vaccines.

Recombinant subunit and epitope-focused vaccines were refined using structure-guided antigen design. Truncated EDIII constructs expressed in *Pichia pastoris* or CHO cells elicit type-specific neutralizing antibodies while avoiding enhancement of implicated epitopes [113,114]. Coupling these constructs to nanoparticle display systems may improve stability and promote targeted responses to conserved epitopes relevant for cross-genotype protection [115]. Taken together, these emerging platforms provide opportunities for rapid antigen updates, improved genotype coverage, and scalable production (Table 3).

### 5.2. Pan-Flavivirus Vaccine Approaches and Cross-Protection Potential

The close antigenic relationship between flaviviruses has motivated the development of broad-spectrum vaccine candidates capable of inducing cross-protection against JEV, DENV, ZIKV, and WNV [125]. These strategies seek to elicit cross-neutralizing antibodies while minimizing the risk of enhancement, which has complicated prior dengue vaccine programs [126].

Structure-guided antigen design has identified conserved epitopes within the E protein fusion loop and domain III lateral ridge as potent targets for cross-reactive antibodies [127]. Cryo-electron microscopy has confirmed that broadly neutralizing human antibodies often recognize quaternary epitopes formed by dimeric rather than monomeric surfaces [54]. Based on these insights, stabilized E dimers or mosaic constructs have achieved simultaneous neutralization of JEV, DENV, and ZIKV in preclinical tests [128].

Hybrid VLPs containing epitopes from multiple flaviviruses elicit cross-reactive neutralizing antibodies without detectable enhancement in vitro and in murine models [129]. Chimeric vaccines using YF-17D or attenuated dengue backbones expressing the JEV prM and E genes provide protection against both homologous and heterologous challenges [130].

Computationally optimized multivalent epitope constructs and nanoparticle formulations broaden immune responses across multiple lineages [131]. These precision-oriented approaches may help balance breadth and safety, enabling protection against diverse JEV genotypes while minimizing the risk of enhancement.

Heterologous primer boosting strategies are promising. Priming with an inactivated JEV vaccine followed by boosting with mRNA or chimeric constructs enhances neutralizing and T cell responses [132]. This approach may be beneficial in regions where overlapping flavivirus exposure shapes the baseline immunity.

Despite encouraging progress, the translation of pan flavivirus candidates remains challenging because the correlates of breadth, durability, and enhancement avoidance are not yet fully defined. Standardized serological assays are required to distinguish between protective and potentially enhancing antibody responses.

### 5.3. Challenges and Future Directions in Vaccine Development

Despite major progress, scientific and operational challenges continue to affect the global JE vaccination strategies. Viral evolution, antigenic drift, waning immunity, and manufacturing constraints remain the central concerns [88,133]. Although licensed vaccines based on GIII strains retain strong real-world effectiveness, multiple studies have reported lower cross-neutralization against GI and GV strains [15,134]. These findings reinforce the need for continuous genomic surveillance and periodic evaluation to justify future vaccine strain updates.

Cross-reactivity and ADE among flaviviruses, although theoretical in JEV vaccination, remain safety considerations in dengue- or Zika-endemic regions, where pre-existing immunity may shape vaccine-induced responses [135,136].

The durability of the protection and optimization of booster policies requires further evaluation. Neutralizing antibody titers may wane within 3–5 years, especially in young children, although memory lymphocyte responses provide residual immunity [88,133]. Improved correlates of protection, including systemic serology and memory cell signatures, may help refine long-term immunity assessments.

Persistent logistical and equity barriers continue to limit access to JE vaccines in low-resource settings. Live-attenuated vaccines require strict cold-chain maintenance, whereas inactivated vaccines are expensive to manufacture. Enhancing the production capacity, developing thermostable formulations, and supporting technology transfer are priorities for equitable vaccination [42,88].

Future innovations will rely on integrating structural vaccinology, computational antigen design, and advanced delivery technologies to broaden the antigenic coverage and improve durability. A health framework that links human, animal, and vector surveillance is essential for anticipating genotype movements and guiding vaccine strategies.

## 6. Therapeutic Approaches

Therapeutic development for JEV includes direct-acting antivirals, monoclonal antibodies, and repurposed agents, reflecting diverse approaches to limit viral replication, reduce neuroinflammation, and improve neurological outcomes. Current therapeutic candidates can be grouped according to the level of supporting evidence. Several compounds exhibit antiviral activity only in cell culture systems and therefore represent early exploratory leads. Others have demonstrated improved survival or reduced viral burden in animal models, which fall into the advanced preclinical stage. A smaller number of agents already have human safety data from use against other viral infections and therefore represent repurposing candidates with more immediate translational potential. Distinguishing these evidence levels provides a clearer framework for interpreting the direct-acting antivirals, monoclonal antibodies, and repurposed or host-directed agents discussed in this section.

### 6.1. Direct-Acting Antivirals

Although vaccination remains the primary strategy for JEV prevention, no licensed antiviral therapy exists, and clinical management remains supportive. Recent advances in molecular virology have facilitated the screening of small-molecule inhibitors that target key stages of the viral replication cycle [137]. These agents remain in the preclinical stages, and their translational potential depends on the improvement of central nervous system penetration and toxicity profiles. Among these candidates, only a few agents have shown improved survival in animal models, and none have progressed to controlled clinical evaluation.

Entry and fusion inhibitors block E protein-mediated attachment and membrane fusion. Peptide analogs derived from the E protein fusion loop, with small molecules such as arbidol and suramin, inhibit viral entry in vitro and protect mice from lethal challenges [138,139]. Agents that interfere with endosomal acidification, including chloroquine, bafilomycin A1, and cathepsin L inhibitors, also reduce fusion efficiency, although their therapeutic windows and central nervous system permeability are limited.

Viral enzyme inhibitors have received particular attention because NS3 and NS5 mediate essential protease, helicase, and RNA polymerase activity. Structure-guided screening has identified benzimidazoles, flavones, and nucleoside analogs with potent in vitro activities [26]. Sofosbuvir, an approved hepatitis C virus polymerase inhibitor, effectively suppressed JEV replication in vitro and in animal models [140]. However, dose requirements, blood–brain barrier penetration, and lack of human data remain major barriers to its clinical translation.

Host-directed antivirals have emerged as a complementary strategy. Compounds targeting GRP78, fatty acid synthase, and autophagy-related kinases (PI3K and mTOR) inhibit viral assembly and reduce replication in neuronal cells [20]. Balancing antiviral potency with host toxicity is a significant challenge, and most candidates remain in the early stages of development. Continued structure-guided optimization and high-throughput screening efforts are expanding the antiviral pipeline, with several advances in preclinical pharmacokinetic evaluations.

### 6.2. Monoclonal Antibody Therapies

Monoclonal antibodies (mAbs) targeting the viral E protein are promising candidates for prophylactic and therapeutic interventions [65]. Their rapid onset of protection and strong neutralizing activity make them attractive emergency countermeasures, although none have progressed to clinical trials. Preclinical studies have demonstrated strong neutralizing activity, but human evaluation has not yet begun.

Epitope mapping revealed neutralizing determinants within the E protein domain III (EDIII) and fusion loop (domain II). Potent mAbs, such as E16, 2H4, and 2F2, stabilize E protein dimers and block the fusion, providing complete protection in murine models [141]. Humanized broadly reactive antibodies, including LZY3412, target the conserved quaternary epitope shared between JEV and ZIKV and neutralize multiple JEV genotypes [142].

Passive immunotherapy significantly reduces viremia, brain viral load, and mortality in animal models, even when administered up to 48 h after infection [143]. Combination mAb regimens targeting non-overlapping epitopes enhance potency and limit virus escape. Fc engineering approaches extend the half-life and modulate Fc gamma receptor interactions to reduce the enhancement risks [144]. However, restricted blood–brain barrier penetration, high production costs, and the absence of clinical data represent substantial translational barriers. Novel bispecific formats and optimized delivery routes may improve therapeutic feasibility.

### 6.3. Repurposed Antiviral Agents

Drug repurposing provides a time-efficient route for identifying candidates with established safety profiles in humans [137]. Nucleoside analogs targeting viral polymerases, including favipiravir and ribavirin, have demonstrated broad anti-flaviviral activities. Favipiravir reduces viremia and mortality in mouse models [145]. Ribavirin inhibits replication by depleting intracellular GTP pools [146], although its therapeutic index in vivo is limited by its toxicity. Combination therapy with interferon-α or additional nucleoside analogs has additive antiviral effects [147]. These agents benefit from existing human safety data, although none have shown confirmed clinical benefits for JE.

Host-targeted repurposed agents include chloroquine and hydroxychloroquine, which interfere with endosomal pH, and mycophenolic acid, which inhibits inosine monophosphate dehydrogenase (IMPDH) and modulates immunity [148]. Other broadly acting agents, such as arbidol, suramin, and ivermectin, target distinct entry or replication pathway [149]. Minocycline combines antiviral activity with neuroprotective effects by reducing microglial activation and cytokine release [150,151]. Therefore, repurposed agents represent practical candidates for rapid progression toward translational testing, although improvements in brain penetration and controlled comparative studies are needed. Currently, no antiviral therapy for JE is licensed.

## 7. Pathogenesis-Targeted and Immunomodulatory Therapies

### 7.1. Neuroinflammation and Cytokine Modulation

The neuropathogenesis of JEV is largely driven by excessive neuroinflammation rather than direct cytopathic injury. Following neuroinvasion, viral replication within neurons and glial cells activates microglia, leading to the release of pro-inflammatory cytokines, such as TNF-α, IL-6, and IL-1β, along with oxidative stress that exacerbates neuronal damage [152,153]. These processes significantly contribute to acute encephalitic injury and long-term sequelae, making host-targeted immunomodulation a rational therapeutic strategy. Current therapeutic candidates can be grouped according to the level of evidence supporting their use. Some compounds have demonstrated antiviral effects only in cell culture systems and, therefore, represent early stage leads. Others have shown improved survival or reduced viral burden in animal models and have reached the advanced preclinical stage. A smaller number of agents have been used clinically for other viral infections and are considered repurposing candidates with potential translational value. These distinctions clarify the realistic maturity of each approach and help identify the most promising options for future developments.

Strategies to reduce microglial activation and cytokine dysregulation have shown neuroprotective effects. Inhibiting pro-inflammatory pathways through TNF-α or IL-6 blockade, activating peroxisome proliferator-activated receptor gamma, or JAK–STAT signaling suppresses cytokine storms and preserves BBB integrity in experimental models [154,155,156]. However, therapeutic timing is critical because excessive immunosuppression may compromise viral clearance and worsen outcomes.

In addition to direct cytokine inhibition, targeting redox and metabolic stress offers complementary neuroprotection. Antioxidants and mitochondrial-stabilizing agents consistently reduce oxidative injury and improve neuronal survival in viral encephalitis models [157,158]. These findings highlight the need to align immunomodulation and metabolic stabilization with antiviral treatment windows to avoid impairing innate viral control while mitigating secondary inflammatory injury. Optimization of dosing schedules and integration of anti-inflammatory strategies with direct-acting antivirals may offer the greatest translational potential.

### 7.2. Neuroprotective and Regenerative Strategies

JEV-associated neurodegeneration is characterized by neuronal loss, synaptic disruption and impaired neurogenesis. Although antiviral and immunomodulatory approaches reduce early injury, restoring neuronal networks and synaptic functions is essential for long-term recovery.

Neuroprotective approaches aim to preserve the mitochondrial integrity, prevent excitotoxicity, and enhance trophic support in the brain. Agents that modulate NMDA receptor activity or enhance neurotrophic signaling, including TrkB receptor agonists and brain-derived neurotrophic factor analogs, improve neuronal survival and dendritic regeneration in experimental models [159,160]. Cell-based and cell-free regenerative strategies are promising approaches to this purpose. Transplantation of neural or mesenchymal stem cells reduces microglial activation, enhances neurogenesis, and improves behavioral outcomes, whereas stem cell-derived exosomes enriched with neuroprotective microRNAs reproduce many of these benefits without the risks of cell transplantation [161,162].

Despite encouraging preclinical results, translation to human therapy remains limited by challenges in delivery across the blood–brain barrier, precise timing of intervention, and long-term safety considerations. Defining therapeutic windows and optimizing minimally invasive delivery are essential for unlocking the regenerative potential of these interventions.

### 7.3. Combination Therapies and Integrated Treatment Models

JEV neuropathogenesis arises from interconnected processes of viral replication, immune-mediated inflammation, and neuronal degeneration. As these mechanisms reinforce each other, monotherapies are unlikely to provide durable benefits. Therefore, integrated therapeutic models that combine antiviral, anti-inflammatory, and neurorestorative interventions represent a rational approach [42,151] (Table 4).

Rather than focusing on specific combinations, this section highlights the conceptual synergy between therapeutic modalities. Antivirals reduce early viral replication, immunomodulators limit secondary inflammatory damage, and regenerative agents promote neuronal repair. Coordinated application of these mechanisms may decrease acute mortality and reduce chronic neurological disabilities.

Emerging system-based and personalized medicine models have further refined this multimodal framework. The integration of systems immunology, pharmacogenomics, and neuroimaging biomarkers may enable patient stratification based on immune status, viral load, and neurodegenerative stage [163,164]. Such precision-guided approaches will help to determine the optimal sequencing and timing of antiviral, anti-inflammatory, and regenerative therapies. Ultimately, a multimodal, patient-tailored treatment strategy that links early viral suppression with targeted immunomodulation and neurorestoration offers the most realistic prospect of reducing morbidity and long-term neurological sequelae patients with JE.

**Table 4:** Therapeutic strategies under Investigation for JEV.

Category	Representative Agents	Molecular Target/Mechanism	Model Efficacy	Translational Limitations	Readiness Level	Ref.
Direct-acting antivirals	Sofosbuvir, Favipiravir, Ribavirin	Inhibit NS5 RdRp; block replication	Reduce viremia; increase survival in mice	Limited CNS penetration; toxicity; human data lacking	In vitro to mouse models	[137,146,165]
Host-targeted antivirals	Arbidol, Suramin, Chloroquine	Block viral entry or endosomal acidification	Inhibit replication in neuronal cells	Narrow therapeutic window; off-target effects	In vitro to early preclinical	[139,166,167]
Immunomodulators	Anti TNF, anti IL-6, JAK–STAT inhibitors	Suppress cytokine storms; modulate immune response	Reduce neuroinflammation and neuronal apoptosis	Risk of impairing viral clearance; timing-dependent efficacy	Mouse models	[168,169]
Neuroprotective agents	Memantine; antioxidants	Block NMDA receptor; reduce oxidative stress	Improve neuronal survival and behavior	Uncertain dosing window; incomplete durability data	Preclinical	[160,170,171]
Stem cell/exosome therapies	MSCs, hNSCs, MSC-derived exosomes	Enhance neurogenesis; repair BBB	Improve motor recovery; reduce inflammation	Delivery challenges, safety concerns; scalability issues	Early preclinical	[159,161]

## 8. Conclusions and Perspectives

Over the past seven decades, vaccination and vector control have transformed JEV from one of the most devastating causes of viral encephalitis in Asia to a largely preventable disease in most endemic countries. However, JEV remains a re-emerging and evolving threat, particularly in regions where ecological changes, climate variability, and uneven vaccine coverage sustain transmission [52]. Recent spatiotemporal analyses have confirmed the northward expansion and establishment of new ecological niches, underscoring the need for continuous genotype-informed surveillance [58].

Advances in molecular virology and immunology have refined our understanding of JEV replication, neurotropism, and immune evasion. Genomic and structural studies have identified genotype-specific mutations in the E protein that influence receptor binding, virulence, and vaccine sensitivity, reinforcing the importance of integration sequencing, phylogeography, and antigenic cartography for variant detection [68]. As genotype I predominates and GV re-emerges, routine antigenic monitoring and real-time genomic surveillance should guide the assessment of vaccine strain alignment and cross-genotype protection.

The widespread deployment of live-attenuated and Vero cell-derived vaccines has substantially reduced disease burden. Nevertheless, waning immunity in some age groups and variable seroconversion after fractional intradermal dosing highlight the need to refine booster policies and long-term protection intervals [109]. Continuous post-marketing monitoring and population-based serosurveillance are essential to sustain herd immunity and identify potential genotype-mismatches.

Next-generation vaccine platforms, including mRNA, DNA, VLPs, and nanoparticle vaccines, offer adaptability, scalability, and improved thermostability compared with traditional vaccines. Several candidates have demonstrated enhanced antigen presentation, strong neutralizing activity, and the capacity for rapid antigen updating, supporting their potential to address genotype shifts. The integration of structure-guided antigen design and advanced manufacturing may ultimately enable a universal, genotype-inclusive vaccine that balances the breadth, safety, and accessibility of the vaccine.

Although prevention remains the cornerstone of control, the absence of licensed antiviral agents has created a critical therapeutic gap. Structure-based drug discovery and repurposing efforts have identified NS3 and NS5 enzyme inhibitors, and nucleoside analogs, such as sofosbuvir and favipiravir, which show preclinical antiviral and neuroprotective activities [151]. Combination strategies that combine direct-acting antivirals with immunomodulatory or neuroprotective agents may provide the most effective means of reducing viral load, inflammation, and neuronal injury. Monoclonal antibodies engineered for improved neutralization breadth and CNS penetration may eventually complement vaccination for prophylactic or postexposure therapies.

Host-directed and regenerative strategies are gaining momentum, beyond viral suppression. Stem cell-derived exosomes, antioxidant adjuvants, and neurotrophic factor analogs show promise for restoring BBB integrity and promoting neurogenesis [159]. Aligning these host-targeted approaches with antiviral and immune-based therapies may shift the treatment goals from survival to functional neurological recovery.

Sustainable JEV control requires an integrated approach rather than an isolated intervention. A multidisciplinary health framework connecting human, animal, and vector surveillance is indispensable for early outbreak prediction, rapid vaccine deployment, and coordinated response. Leveraging genomic surveillance, climate-based forecasting, and AI-driven vector modeling will enhance preparedness in endemic and newly affected regions [172]. Simultaneously, ensuring equitable global access through regional manufacturing, technology transfer, and improved cold-chain systems is essential. Ultimately, the convergence of next-generation vaccine platforms, host-directed therapeutics, and integrated One Health surveillance provides the most realistic path toward the long-term control and potential elimination of JE virus infection.

## Figures and Tables

**Table 1:** Comparison of genotype-specific PRNT reduction and available field or experimental evidence.

Genotype	Reported PRNT Reduction Compared with GIII Derived Vaccines	Field or Experimental Evidence
GI	Approximately two-fold reduction reported.	High vaccine effectiveness is still observed with no clear increase in breakthrough infections.
GIV	Limited data suggest a modest reduction in neutralizing titers, although the precise fold reduction has not been consistently quantified.	No consistent evidence of higher infection rates among vaccinated populations.
GV	More than four-fold reduction reported in several studies.	Some animal studies report loss of neutralization or reduced protection. Human field data remain very limited.

PRNT, plaque-reduction neutralization tests.

**Table 3:** Emerging vaccine platforms and their relevance for cross-genotype protection.

Platform	Antigen Design/Genotype Relevance	Expression System	Immune Profile/Protection	Ref.
mRNA vaccine	E or EDIII mRNA (GI or GIII); rapid antigen updating enables fast response to genotype I and genotype V emergence	LNP-encapsulated mRNA	Strong neutralizing antibodies; balanced Th1 and Th2; complete protection in mice with GI-matched constructs	[105,106]
DNA vaccine	prM–E with molecular adjuvants; can incorporate genotype-matched inserts to enhance breadth	Plasmid DNA via electroporation	Humoral and cellular responses; moderate NAb titers; requires delivery optimization for improved cross-genotype coverage	[116,117,118,119]
VLP	prM + E; preserves quaternary epitopes important for cross-genotype neutralization including against GV	Baculovirus or mammalian systems	Strong neutralizing responses; complete protection in murine models; enhanced recognition of conformational epitopes	[112,120,121,122]
Viral-vector vaccine	prM–E expressed from recombinant adenovirus, measles, or vaccinia; flexible backbone allows rapid insertion of genotype-updated sequences	Adenovirus, measles, vaccinia vectors	Robust antibody and CD8^+^ responses; broad cross-reactivity	[70,120,121,122,123]
Recombinant subunit/epitope-focused vaccine	EDIII or NS1 constructs; epitope focusing avoids ADE-associated regions and targets conserved epitopes shared across genotypes	*Pichia pastoris*/CHO cells	Type-specific and cross-neutralizing antibodies; reduced enhancement risk	[40,124]

LNP, lipid nanoparticle; prM–E, precursor membrane and envelope proteins; VLP, virus-like particle; EDIII, envelope protein domain III; NS1, nonstructural protein 1; ADE, antibody-dependent enhancement.

## Data Availability

No new data were created or analyzed in this study. Data sharing is not applicable to this article.
